# Spontaneous jumping, bouncing and trampolining of hydrogel drops on a heated plate

**DOI:** 10.1038/s41467-017-01010-8

**Published:** 2017-10-13

**Authors:** Jonathan T. Pham, Maxime Paven, Sanghyuk Wooh, Tadashi Kajiya, Hans-Jürgen Butt, Doris Vollmer

**Affiliations:** 1 Max Planck Institute for Polymer Research, Ackermannweg 10, 55128 Mainz, Germany; 2 Present Address: Department of Chemical and Materials Engineering, University of Kentucky, 177F. Paul Anderson Tower, Lexington, KY 40506 USA; 3 Present Address: School of Chemical Engineering & Materials Science, Chung-Ang University, 84 Heukseok-ro, Dongjak-gu, Seoul 06974 Korea; 4 Analysis Technology Center, Fujifilm R&D, 210 Nakanuma, Minamiashigara, Kanagawa 250-0123 Japan

## Abstract

The contact between liquid drops and hot solid surfaces is of practical importance for industrial processes, such as thermal spraying and spray cooling. The contact and bouncing of solid spheres is also an important event encountered in ball milling, powder processing, and everyday activities, such as ball sports. Using high speed video microscopy, we demonstrate that hydrogel drops, initially at rest on a surface, spontaneously jump upon rapid heating and continue to bounce with increasing amplitudes. Jumping is governed by the surface wettability, surface temperature, hydrogel elasticity, and adhesion. A combination of low-adhesion impact behavior and fast water vapor formation supports continuous bouncing and trampolining. Our results illustrate how the interplay between solid and liquid characteristics of hydrogels results in intriguing dynamics, as reflected by spontaneous jumping, bouncing, trampolining, and extremely short contact times.

## Abstract (web summary)

Drops of liquid on a hot surface can exhibit fascinating behaviour such as the Leidenfrost effect in which drops hover on a vapour layer. Here Pham et al. show that when hydrogel drops are placed on a rapidly heated plate they bounce to increasing heights even if they were initially at rest.

## Introduction

When water is splashed onto a hot pan, drops of water bead up and smoothly glide along the surface. This common kitchen observation arises from the so-called Leidenfrost effect where drops are separated from the surface by a film of their own vapor^1–5^, first discovered by Leidenfrost in 1756. The surface temperature must be higher than the Leidenfrost temperature, such that the water vaporizes sufficiently fast to form an insulating vapor layer underneath the drop. Below the Leidenfrost point, drops make contact with the surface, which is essential for cooling (i.e., spray cooling) because it provides rapid heat transfer^6–8^. In contrast, the vapor layer in the Leidenfrost effect produces adhesion-free drops, which enables drop bouncing^2^ and easy transportation of objects over a surface^9^. Vapor-mediated bouncing also occurs on superhydrophobic surfaces^10^, on hydrophilic surfaces with a low velocity impact^11, 12^, and on solid surfaces undergoing sublimation^10^.

Recently, water drops resting on a superhydrophobic surface have even been demonstrated to spontaneously jump and start trampolining upon rapidly reducing the background pressure^13^. Trampolining is characterized by a restitution coefficient (*e*) higher than one; that is, the drop bounces to a higher height than the initial height. Typical bouncing, on the other hand, is characterized by drops rebounding from a surface with a restitution coefficient equal to or below one. Upon this background pressure reduction, jumping, bouncing and trampolining are driven by vapor pressure buildup within texture of the superhydrophobic surface under the drop. In addition, water drops have also been shown to jump from surfaces by other mechanisms. For example, drops at ambient temperature on a superhydrophobic surface jump during coalescence due to a gain in surface energy^14–16^. A similar take-off also occurs when a drop, already in the Leidenfrost state, becomes very small^17^.

During bouncing (or trampolining) of a water drop on a stiff surface, kinetic energy is converted to surface energy. That surface energy is reconverted into kinetic energy during the subsequent retraction and rebound^18–21^. When a water drop is impacting an elastically compliant surface, part of the total energy is reserved for the elastic energy required to deform the surface, which affects the contact time, drop deformation, and coefficient of restitution^22, 23^. In contrast to a liquid drop, during bouncing of a soft solid ball on a stiff surface, kinetic energy is converted to elastic energy before being reconverted into kinetic energy. In reality, much of the kinetic energy is lost during impact due to friction or adhesion^24^. After a couple of bounces, a ball usually comes to rest. It is therefore surprising to find that when hydrogel balls are dropped onto a hot pan, they bounce for an extended period of time, which can be on the order of several minutes (https://www.youtube.com/watch?v=OfcCsP-T1pc)^25^. A hydrogel is an elastic, highly-water-filled polymer network in which the elasticity and the water content are tunable by the crosslinking density. Although hydrogels can be over 99% water^26^, they display soft solid characteristics. Hence, hydrogels are an ideal material to investigate the interplay between liquid and solid aspects of heat-induced jumping and bouncing.

Here, we show that the combination of elastic deformation and fast evaporation of water in hydrogel drops gives rise to unique dynamics on a superheated surface. The hydrogels, initially at rest, spontaneously jump from a rapidly heated surface and subsequently bounce and trampoline as many as 100 times. In the following, we define jumping as the transition from contact to non-contact when the hydrogel is moving in the vertical direction. By varying wettability of the surface and modulus of the hydrogel, we show that spontaneous jumping is most favorable when the surface is hydrophilic and the hydrogel-surface adhesion is low. Subsequent bouncing and trampolining is governed by hydrogel drop elasticity and fast water vaporization above the Leidenfrost temperature while very short contact times are observed below the Leidenfrost temperature.

## Results

### Preparation of millimetric hydrogel drops

Millimeter-scale hydrogel drops are fabricated by gently placing 10 microliters of an acrylamide/methylenebisacrylamide (AAm/BAAm) monomer/crosslinker solution on a soot-templated, superamphiphobic, concave surface (Supplementary Fig. 1)^27, 28^. The superamphiphobic candle-soot surface offers a high contact angle, allowing hydrogel drops to be prepared upon UV-initiated polymerization. During polymerization, the drops are rolled to achieve sphericity (Supplementary Movie 1). The nearly spherical drops of radius *R* ≈ 1.25 mm are then rolled off the surface and stored in excess water for at least 48 h. The modulus (*E*) of the hydrogels is modified by changing the ratio of monomer and crosslinker (Methods section), leading to *E* = 2, 25, and 320 kPa.

### Heat-induced hydrogel drop jumping

**Fig. 1:**
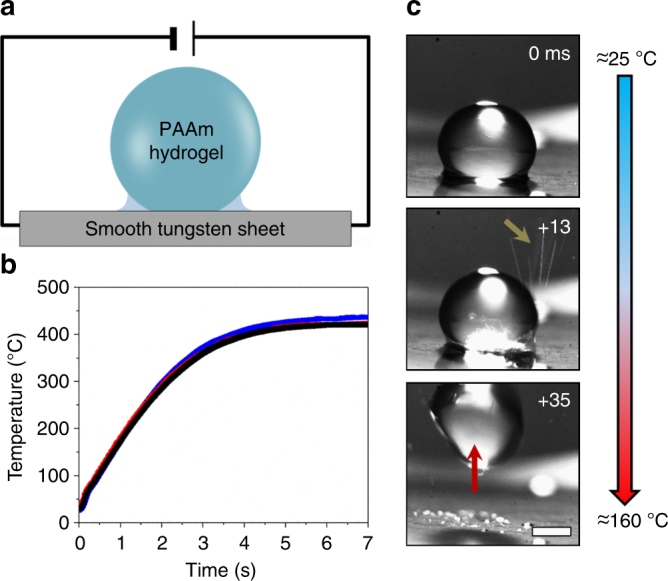
Jumping hydrogel drops from a heated surface. **a** Schematic of heating and jumping experimental setup where a polyacrylamide (PAAm) hydrogel drop (diameter ≈ 2.5 mm) is placed on a tungsten sheet. A water meniscus surrounds the hydrogel. An electric current is applied to heat up the plate. **b** A temperature–time profile obtained by an IR camera for three independent measurements at a current of 105 A. **c** Experimental observations of a hydrogel drop jumping from the surface upon heating (Supplementary Movie 2). Bubbles burst and small droplets are observed to eject from the side (+13 ms, arrow) before the hydrogel jumps upward (+35 ms). The 0 ms frame is taken arbitrarily to illustrate the timescale of the event. Scale bar: 1 mm

**Fig. 2:**
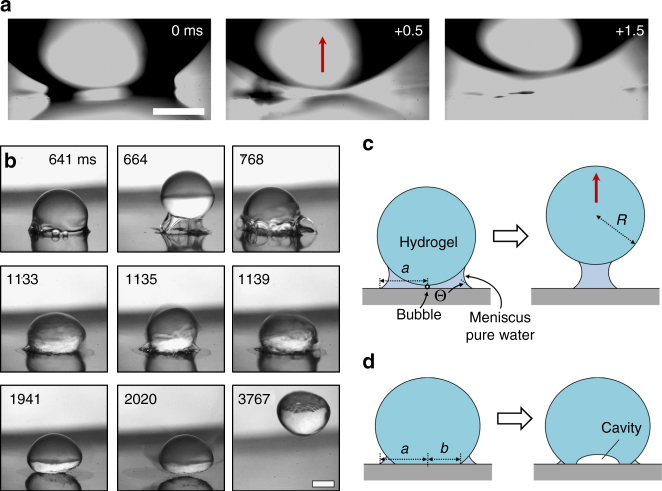
Hydrogel drop jumping due to vaporization of the meniscus. **a** Time snapshots of the jumping of a 25 kPa hydrogel drop (Supplementary Movie 3). The meniscus is clearly visible in the first image and is completely gone 0.5 ms later after meniscus explosion. The 1.5 ms image illustrates the upward motion of the hydrogel. Scale bar: 0.5 mm. **b** Evolution of a 2 kPa hydrogel drop jumping after burning. Bubbles first form underneath the drop (Supplementary Movie 4). The hydrogel drop attempts to jump (664 ms), but adhesion by fibrils holds it back. The drop remains on the surface and rapid vibrations are observed (1133–1139 ms). The polymer fibrils start to burn away and the drop is released from the surface (2020 ms). Scale bar: 1 mm. Illustrations for the proposed mechanism of (**c**) jumping of the 25 kPa hydrogel in part (**a**) and (**d**) cavity formation and burning of the 2 kPa hydrogel in part (**b**)

A 25 kPa hydrogel drop is placed on a smooth tungsten sheet (RMS roughness 15 ± 5 nm, water contact angle less than 5°, Supplementary Figs. 2 and 3) at ambient temperature and pressure, and a meniscus is acquired from water that swells the gel (Fig. 1a)^29^. After 30 s, the tungsten surface heats quickly (150 ± 5 °C s^−1^ from 0 to 1 s) to a maximum temperature of 430 ± 10 °C upon applying a current of 105 A. The temperature–time profile is determined using an infrared camera (Fig. 1b and Supplementary Fig. 4). In this particular experiment, after 500 ms (at ≈100 °C), the drop begins to slightly vibrate on the surface while being held in place by capillarity. This is associated with boiling where bubbles form in the water meniscus zone and release into the atmosphere. Boiling also leads to the ejection of small droplets outward from the contact zone with typical diameters of 50–100 µm and speeds up to 3 m s^−1^ (Fig. 1c, 13 ms). The hydrogel drop then jumps from the surface (Fig. 1c, 35 ms), driven by an explosion-like release of vapor under the drop. Remarkably, jumping heights can be as high as ~15 mm, or 6× the drop diameter. As shown in Fig. 2a (Supplementary Movie 3), the water meniscus completely disappears in ≲0.5 ms at the onset of jumping. While these general events are consistently observed, the jumping time can vary. Over 18 experiments, the jump occurs at 900 ± 150 ms, which is at a temperature of ≈160 °C.

Intuitively, one expects the modulus of the hydrogel to play a role in jumping. To study the effect of hydrogel modulus, we conducted the same heating experiments with a softer 2 kPa hydrogel. In contrast to the 25 kPa hydrogels, jumping due to an explosion-like event is not observed. Adhesion between the hydrogel and the surface is much higher (Supplementary Movie 4), leading to delayed jumping. Bubbles still form under the drop and the hydrogel attempts to jump (Fig. 2b). However, thin fibrillary bridges, a common characteristic in soft adhesives^30–32^, inhibit release of the hydrogel from the surface (Fig. 2b, 664 ms). The hydrogel then begins to vibrate vigorously (Fig. 2b, 1133–1139 ms). As the heated plate exceeds ≈300 °C, the fibrillary bridges be﻿gin to burn (Fig. 2b, 2020 ms) and the drop is released from the surface. This delayed jumping is followed by bouncing and trampolining (Fig. 2b, 3767 ms). It is important to discriminate between the two types of jumping. We define the first explosion-like jumping as meniscus jumping and this second adhesive jumping as jumping after burning.

On the other spectrum, when a 320 kPa hydrogel is used, jumping can occur (Supplementary Movie 5) but with poor reproducibility. This is likely associated with the small contact area of a stiff–stiff contact accompanied by easy vapor release radially from the contact zone. Therefore, in the present study we focus on the 25 and 2 kPa hydrogels.

### Spontaneous jumping mechanism

Our observations point toward a rapid release of vapor from the meniscus as the key mechanism for hydrogel drop jumping. We hypothesize that pressure released from a bubble underneath the drop propels it in the upward direction while gravity, capillary adhesion from the meniscus, and the polymer-surface interfacial adhesion act in the opposing direction. To describe jumping in a simplified manner, the energies are balanced as *U*_bubble_ = *U*_gravity_ + *U*_capillary_ + *U*_polymer_. Energy stored in a vapor bubble, which has formed underneath the hydrogel due to boiling, can be written as *U*_bubble_ = (*P*−*P*_0_)*V*_bub_. The gravitational energy is given by *U*_gravity_ = *ρghV*_gel_. Here *P* is the pressure in the bubble, *P*_0_ is the atmospheric background pressure, *V*_bub_ is the bubble volume, *ρ* is the hydrogel density, *g* is the acceleration of free fall, *h* is the jump height and *V*_gel_ = (4/3)*πR* ^3^ is the volume of the hydrogel drop. ${U_{{\rm{capillary}}}} = \pi {a^2}\gamma (1 + \cos {\rm{\Theta }})$ is an estimate for the energy required to move the hydrogel normal to the solid surface until the capillary bridge ruptures (Fig. 2c, Supplementary Note 1), where *a* is the contact radius of the capillary meniscus and *γ* is the surface tension of water. Our experiments show that rupture of the capillary bridge occurs due to an explosion-like release of vapor, rather than by simply pulling the hydrogel up. Thus our calculation is only an approximation for the energy associated with breaking the capillary bridge. *U*_polymer_ = *πb* ^2^ *w* is the work required to detach the polymer from the surface, where *b* is the contact radius of the hydrogel and *w* is the work of adhesion per unit area (Fig. 2d). By rearrangement of the energy balance, the expected jump height is estimated as:

1 $$h \approx \frac{{\left( {P - {P_0}} \right){V_{{\rm{bub}}}} - \pi {a^2}\gamma (1 + \cos {\rm{\Theta }}) - \pi {b^2}w}}{{\rho g{V_{{\rm{gel}}}}}}$$

When placing a hydrogel drop onto the hydrophilic tungsten surface, a water meniscus of contact radius *a* is formed. One consequence of heating is evaporation of the meniscus, decreasing the contact radius *a* and hence the capillary attraction.

To compare our experiments to Eq. 1, we consider meniscus jumping of the 25 kPa hydrogel. The 2 kPa hydrogel displays complex adhesion and burning that cannot be simply described. For the 25 kPa hydrogel, polymer adhesion plays little role (Fig. 2a), leaving capillary energy as the adhesion term. Between 150 and 170 °C, *P* = 480–790 kPa and the atmospheric pressure is *P*_0_ = 101 kPa^33^. Assuming we have one spherical bubble confined under the hydrogel (Fig. 2c), the volume is approximated by optical images of the meniscus height directly before jumping (120 µm) as an upper bound, giving *V*_bub_ ~ 10^−12^ m^3^. Taking *P*−*P*_0_ = 520 kPa (at 160 °C), *γ* = 59 mN m^−1^ (at 100 °C), and a typical value of *a* = 0.5 mm, the jump height is calculated to be 5 mm. Consistent with this value, we observe jump heights ranging from a 3 mm up to 14 mm. Variations in experimental observations of the jump height are associated with challenges in measuring the exact bubble size, instantaneous temperature, amount of meniscus evaporation and directionality of the force from the rapid release of vapor. For example, if two bubbles of the same size cause jumping, the height doubles to 10 mm or if the temperature is 180 °C, *P*−*P*_0_ = 900 kPa and the expected jump height is 10 mm, which are both within our observed range.

### Effects of surface wettability and adhesion

**Fig. 3:**
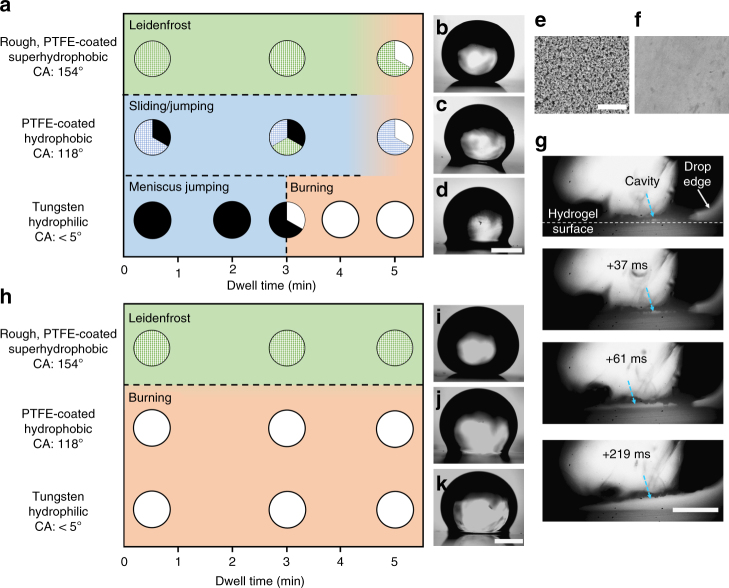
Effects of surface wettability, dwell time, and modulus on jumping. **a** Map of outcomes for heating of 25 kPa hydrogels on **b** superhydrophobic, **c** hydrophobic, and **d** hydrophilic surfaces with corresponding optical images before heating. Data are denoted as meniscus jumping (filled), jumping after burning (unfilled), sliding (blue grid), and Leidenfrost (green diamond). SEM images of (**e**) PTFE-coated TiO_2_ surface and (**f**) unmodified tungsten surface. Scale bar: 3 µm. **g** Zoom in of 25 kPa hydrogel and hydrophilic tungsten interface being burned away (greater than 3 min dwell time). The white spot in the center of the hydrogel is reflection from light. The white dotted line is the interface between the hydrogel and surface. A cavity (dashed blue arrows) initiates and as the plate temperature continues to increase, the interface forms a vapor layer that separates the drop from the surface. Scale bar: 0.5 mm. **h** Map of outcomes for 2 kPa hydrogels on **i** superhydrophobic, **j** hydrophobic, and **k** hydrophilic surfaces with corresponding optical images before heating. Scale bar: 1 mm

To test the adhesion terms in Eq. 1, we investigate the effect of surface wettability and polymer-substrate adhesion on jumping (Fig. 3a–d). Surface wettability controls the contact angle Θ and the contact radius *a*, effectively changing the capillary energy, $\sim {a^2}\gamma (1 + \cos {\rm{\Theta }})$. Since a hydrogel is mostly water, capillary adhesion is expected to decrease as the surface is transformed from hydrophilic to hydrophobic to superhydrophobic. Therefore to investigate the influence of wettability, we prepared hydrophobic polytetrafluoroethylene (PTFE) coated surfaces and superhydrophobic PTFE-coated TiO_2_ particle surfaces (Fig. 3e, f). The work of adhesion *w* increases as a function of dwell time (i.e., duration a drop is left on the surface before heating) for soft materials^34, 35^. Favorable polymer-substrate interactions also drive deformation of the soft hydrogel to create a larger contact, *b* ^36, 37^. Thus this adhesion term is tested by controlling the dwell time.

With a 30 s dwell time, meniscus jumping of 25 kPa hydrogels is always observed on the hydrophilic surface (filled data, Fig. 3a). However it becomes less likely on a hydrophobic surface and is never observed on a superhydrophobic surface. Although jumping can occur on a hydrophobic surface, it is more common that the drop slides over the surface (blue grids, Fig. 3a). Sliding is defined as the drop moving on the surface but still in contact by a water meniscus (Supplementary Movie 6). Likely, a nanoscale vapor film forms on the hydrophobic surface^38^, allowing for release of pressure. For a superhydrophobic surface, the drop goes into a Leidenfrost state (green diamonds) and hovers over the surface (Supplementary Movie 7). The contact area is small such that vapor can be easily released unde﻿rneath the drop.

To investigate the influence of polymer adhesion on jumping, the dwell time was increased from 30 s up to 5 min. On the hydrophilic surface, a transition from meniscus jumping to jumping after burning (open data) is observed at a 3 min dwell time (for *E* = 25 kPa). Upon local burning of the interface, cavities form underneath the drop which fill with vapor, affording regions of zero adhesion (Fig. 3g); the hydrogel and the surface are separated by vapor. Once the polymer fibrils are removed along the entire interface, the drop is released from the surface (Fig. 3g, 219 ms). We note that in the burning regime, bubbles sometimes grow into the hydrogel; the elastic energy to expand into the drop is lower than that of breaking the interface (Supplementary Note 2)^39^. On the hydrophobic and superhydrophobic surfaces, there is a similar trend towards burning as a function of increasing dwell time. In the Leidenfrost regime, occasionally the drop is initially adhered to the surface by small polymer fibrils. When these small adhesion points are released, the drop goes into a Leidenfrost state (Supplementary Movie 7) and pressure is released through an air layer.

We conducted the same set of experiments with the 2 kPa hydrogels (Fig. 3h–k). We never observe meniscus jumping, consistent with our observations in Fig. 2b. For hydrophilic and hydrophobic surfaces, the drop is released only after burning. On a superhydrophobic surface, Leidenfrost hovering is observed for all dwell times. Even though *a* and *b* are small, which leads to low adhesion, jumping does not occur because vapor escapes from the small contact zone. Our results on different wetting surfaces and increasing dwell times illustrate that for meniscus jumping, pressure must be confined underneath the hydrogel while the polymer-surface adhesion must also be low.

### Bouncing and trampolining after jumping

After a drop jumps from the surface, the hydrogel continues to bounce. We separate bouncing by the two types of jumping: bouncing after meniscus jumping and bouncing after burning. We note that bouncing is only investigated on the unmodified, hydrophilic tungsten surfaces because the hydrophobic coating molecules start decomposing around 350 °C.

**Fig. 4:**
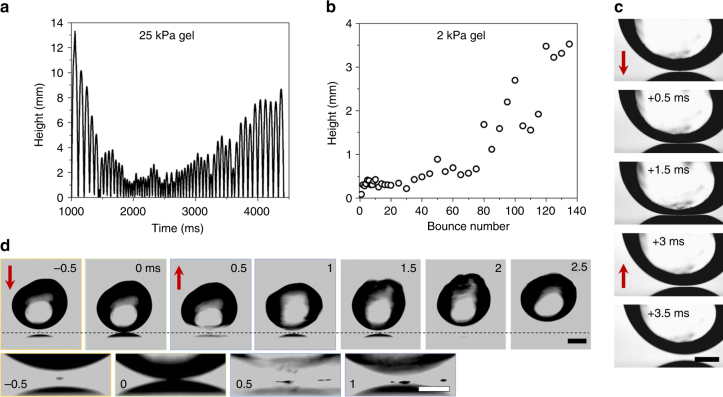
Subsequent bouncing and trampolining. **a** Height vs. time for a 25 kPa hydrogel drop after the initial jump illustrating subsequent bouncing (Supplementary Movie 8). After 4.5 s, the drop has bounced outside the field of view. **b** Height vs. bounce number for a 2 kPa hydrogel after burning the interface (Supplementary Movie 4). **c** An example of Leidenfrost type contact with an impact velocity of *v* ≈ 0.15 m s^−1^ and a surface temperature ≈400 °C. A vapor layer is visible underneath the hydrogel separating the drop from the surface. Scale bar: 0.5 mm. **d** An example of a pressure-induced pulse type contact (Supplementary Movie 9) of a hydrogel with an impact velocity of *v* ≈ 0.25 m s^−1^ and a surface temperature ≈190 °C. Time between images is 0.5 ms. Red arrows illustrate direction of the hydrogel. Scale bar: 1 mm. Insets are zoom-ins of contact zone with labeled times. Scale bar: 0.5 mm

First consider the meniscus jumping of a 25 kPa hydrogel (bottom left of Fig. 3a); after the initial jump, the hydrogel can bounce and trampoline several times (Fig. 4a, Supplementary Movie 8). The amplitude of subsequent bounces decreases before increasing after ~2 s. In general, the amplitude first decreases when the first jump exceeds several mm. When the initial jump is only a few mm, the drop bounces with an approximately constant height before trampolining. After 2 s, the temperature reaches 292 ± 5 °C, at which point trampolining ensues. Trampolining continues until the hydrogel has bounced out of the imaging frame at 4.5 s (at 410 ± 10 °C). To confirm that increased bouncing heights are not due to a significant loss in mass, we measured a negligible change in size of the hydrogel drop during bouncing and trampolining (Supplementary Fig. 5), which is consistent with our calculation of drop evaporation rate (Supplementary Note 3).

In the burning regime, the drop jumps after the interface is thermally degraded (Fig. 2b). A plot of bounce height vs. bounce number reveals a trampolining effect. A characteristic trend is that the bouncing height increases monotonically once the drop is released from the surface (Fig. 4b, Supplementary Movie 4). The release occurs typically between 2 and 3.5 s (corresponding to ~ 300 to 400 °C, which exceeds the dynamic Leidenfrost temperature)^4^. As this period varied, we plot height vs. bounce number to document restitution coefficients greater than one (Fig. 4b).

### Apparent contact time

To understand the impact dynamics, we consider the apparent contact time of bouncing hydrogel drops, which is ≈ 0.5–3.5 ms for the 25 and 2 kPa hydrogels. We first compare the apparent contact time with a Hertzian contact model to determine if impact is elastically described^40, 41^. Measurements of tangent delta ≲0.1 from shear rheology imply that the hydrogel drop behaves like an elastic ball (Supplementary Fig. 6). Thus assuming the hydrogel is an incompressible (i.e., Poisson’s ratio is *ν* = 0.5) elastic solid, the force is given by *F* = (16/9)*ER* ^1/2^ *δ* ^3/2^, where *δ* is the normal compression of the hydrogel. By integration, the elastic energy is:

2 $${U_{{\rm{Elastic}}}} = {\int} {F{\rm d}\delta } = \frac{{32}}{{45}}E{R^{1{\rm{/}}2}}{\delta ^{5{\rm{/}}2}}$$

Equating the kinetic energy, *U*_kin_ = *mv* ^2^/2, with the elastic energy gives the compression as *δ*_Elastic_ = (45*mv* ^2^/64*ER* ^1/2^)^2/5^, where *m* = *ρV*_drop_ is the mass of the drop and *v* is the impacting velocity. The apparent contact time is described by the deformation and the incident velocity as:^41, 42^

3 $${t_{\rm{Elastic}}} \approx 2.9\left( {\frac{{{\delta _{\rm{Elastic}}}}}{v}} \right) = 2.9{\left( {\frac{{15\pi \rho }}{{16E}}} \right)^{\!2{\rm{/5}}}}\frac{R}{{{v^{1{\rm{/5}}}}}}$$

For 25 kPa hydrogel drops colliding at a typical velocity of *v* ≈ 0.2–0.3 m s^−1^, the calculated apparent contact time is *t*_Elastic_ ≈ 2 ms while for the 2 kPa hydrogel, *t*_Elastic_ ≈ 6 ms.

During bouncing, two possible impact types are observed. The first is a Leidenfrost impact where a vapor layer separates the hydrogel from the hot surface, as demonstrated in Fig. 4c. Since there is no direct contact between the hydrogel and surface, we describe the impact with an apparent contact time. In this case, the experimental apparent contact time is ≈3 ms for a 2 kPa hydrogel, which is overestimated by Eq. 3 by a factor of two. This overestimation may result from an increase in the elastic modulus during heating and inhomogeneous evaporation in the burning jumping regime. For a 25 kPa hydrogel, the apparent contact time is in good agreement with elastic contact.

The second impact is characterized by a much shorter contact time of ≲0.5 ms, driven by a pressure-induced pulse. In Fig. 4d, snapshots of a hydrogel drop with this impact type are presented (Supplementary Movie 9). As the drop approaches the surface, a quick pulse pushes the bottom of the hydrogel in the opposing direction. This pressure-pulse is not well described by elastic impact and observed contact times are at least four times shorter than expected by Eq. 3. In contrast to the Leidenfrost impact, high speed imaging illustrates that true contact is made with the surface. As shown by the insets in Fig. 4d, residual water or hydrogel remains on the surface after the bouncing event.

## Discussion

It is instructive to compare the present work to that of Schutzius et al^13^. In their case, a water drop resting on a superhydrophobic surface spontaneously jumps when the background pressure is quickly reduced, driven by pressure development underneath the vaporizing drop. In our case, vapor is similarly produced at the interface of the hydrogel-metal surface, which comes in the form of bubbles in the meniscus. In contrast to their jumping, our meniscus jumping is driven by an explosive loss of the water meniscus, leading to initial jump heights as high as ~15 mm. In the burning regime, the meniscus is already evaporated at the onset of jumping and the hydrogel more closely resembles their water drop in reduced pressure^13^. However unlike water drops, the hydrogels initially adhere to the surface which delays the onset of jumping and bouncing. Once the hydrogel has jumped from the surface after burning, it starts trampolining.

When compared to a pure water drop impacting a superheated surface, hydrogels display shorter (apparent) contact times and smaller deformations. Since hydrogels are elastic materials, we compare our (apparent) contact times with Weisensee et al^22^. They found that an elastically compliant surface can produce a springboard effect and consequently shorten contact times. Like most water drop impacts, the drop spreads. While in that spread geometry, it is pushed off the surface due to the substrate compliance. However, since energy during impact is mostly converted to surface energy, the contact times are still relatively long (~ 6–14 ms), exceeding the shortest contact time of our hydrogel drops by an order of magnitude.

Similar to water drops bouncing on superhydrophobic surfaces in reduced pressure^13^, we attribute a vapor layer underneath the hydrogel drop to an extra pressure allowing for trampolining (Fig. 4a). The rate of mass loss of an evaporating water drop is estimated as:^2^

4 $$\dot m \approx \frac{{\lambda \left( {{T_{\rm{S}}} - {T_{\rm{B}}}} \right)}}{{Lh}}{l^2}$$

where *λ* is the thermal conductivity of vapor, *T*_S_ is the temperature of the substrate, *T*_B_ = 100 °C is the boiling temperature, *L* is the latent heat of vaporization, *h* is the thickness of the vapor film, and *l* is the apparent contact radius of the hydrogel. By Eq. 4, one expects that the mass loss scales linearly with increasing temperature (i.e., *T*_S_−*T*_B_). Assuming the other variables are constant, this produces more available vapor to push the hydrogel upwards. Consistent with this concept, the amplitude starts to plateau around 4 s where the temperature ramp levels off (Figs. 1b and 4a).

For a pure water drop contacting a surface, the drop significantly deforms laterally and a dimple zone of high pressure develops under the drop. Recently, Shirota et al. illustrated that the contact radius at first impact is approximately *R*/3 with a central zone of zero contact (i.e., the dimple)^20^. For the case of hydrogels, the contact is roughly a point contact with a radius less than *R*/10 (Fig. 4d). Elasticity prevents lateral spreading of the hydrogel, greatly reducing the formation of a pressure filled dimple. Instead, that pressure is transferred to the hydrogel by a quick pulse and a wave moves through the drop (Supplementary Movie 9). This pressure-induced shock wave leads to extremely short contact times.

**Fig. 5:**
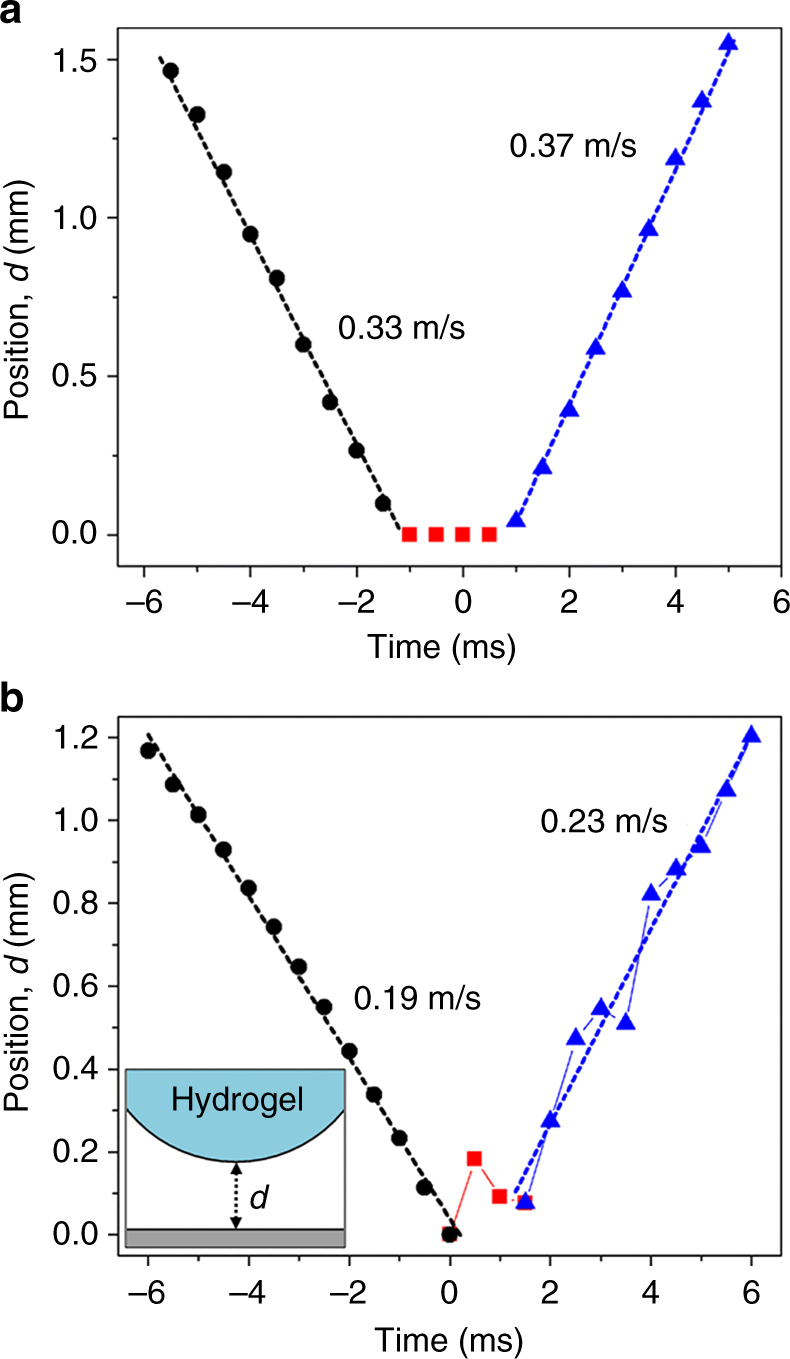
Incoming and outgoing velocities during impact. **a** Position of the bottom of a drop from the surface vs. time for a 25 kPa hydrogel drop impacting the surface at ≈365 °C. The black circles, red squares and blue triangles indicate incoming, contact, and outgoing portions of the impact. **b** Position vs. time for a 25 kPa hydrogel drop impacting the surface at ≈190 °C (Supplementary Movie 9) Inset: schematic illustrating the definition of the position *d*

Our experiments show hydrogel trampolining at temperatures above ≈300 °C (Fig. 4a). For water drops, the transition from contact to Leidenfrost boiling is in the range of 300–400 °C^4^ at a similar Weber number, We = 2*Rρv* ^2^/*γ*. This transition, combined with the increased mass loss expected by Eq. 4, suggests that trampolining only occurs in the Leidenfrost regime. Since the hydrogel bounces in and out of focus, our resolution is insufficient to prove this experimentally. By observing the bottom position of the drop as a function of time for a trampolining hydrogel above 300 °C, we find that indeed the outgoing velocity is higher than the incoming one, in line with this requirement for trampolining (Fig. 5a). Interestingly, a similar measurement on the pressure-pulse impact can also display trampolining (Fig. 5b). In contrast to Leidenfrost bouncing, the bottom of the drop does not move monotonically upwards from the surface because a wave moves through the drop (Fig. 5b, Supplementary Movie 9). While it is clear that trampolining consistently occurs in the Leidenfrost regime, we are not able to strictly limit trampolining to temperatures above the Leidenfrost temperature. Trampolining likely occurs for the pressure-pulse bouncing regime when the initial jump height is less than a few mm.

In summary, we have demonstrated that jumping, bouncing and trampolining are governed by the hydrogel elastic modulus and adhesion, surface wettability, and surface temperature. For meniscus jumping, a water meniscus is required in combination with a hydrophilic surface. Counterintuitively at first glance, hydrogels cannot jump from superhydrophobic surfaces because vapor escapes from the small contact zone. During bouncing, adhesion is effectively zero. This near-adhesionless impact behavior combines with fast vapor production under the hydrogel to allow for continuous bouncing and for trampolining. Notably, the trampolining effect is found on smooth tungsten surfaces where pressure buildup within the surface roughness is not possible. Since hydrogels are elastic, they are not able to deform to the extent of water drops and thus display highly reduced (apparent) contact times.

## Methods

### Chemicals and materials

The following chemicals were purchased from Sigma-Aldrich: Tetraethyl orthosilicate (TEOS, 98%, Germany), trichloro(1H,1H,2H,2H-perfluorooctyl)silane (PFOTS, 97%, USA), Acrylamide (AAm, ≥99%, China), D-glucose (G, ≥99%, Germany), glucose oxidase from aspergillus (GOx, 10 KU, UK), 2-hydroxy-4′-hydroxyethoxy-2-methylpropiophenone (Irgacure 2959, 98%, USA), absolute ethanol (Germany), TiO_2_ nanoparticle paste (diameter: ≈250 nm, WER2-O), and fluorocarbon solvent (Fluorinert FC-770). N,N′-methylenebis(acrylamide) (BAAm, ≥99%) was obtained from Alfa Aesar, Germany. Toluene (ACS grade) and ammonium hydroxide aqueous solution Normapur (28%) were obtained from VWR, France. Acetone (AR grade) was purchased from Fischer Scientific, UK. Amorphous Teflon fluoropolymer (AF1600X) was purchased from DuPont, USA. Water was purified by a Sartorius Arium 611. Paraffin candles were obtained from Real-Handels GmbH, Germany (TIP Haushaltskerzen, 100% paraffin, wick: 100% cotton). 3-well concavity slides (1.4–1.6 mm thick) are from ESCO–Erie Scientific Co., USA). Tungsten sheets (65 × 20 × 0.1 mm) were supplied from Umicore thin film products AG, Germany. All reagents were used as received.

### Superamphiphobic surfaces for hydrogel drop fabrication

3-well concavity slides were coated with a superamphiphobic, soot-templated coating as described below^27, 43^. The slides were cleaned by sonication in toluene, acetone, and ethanol for 5 min, respectively and were then activated by oxygen plasma (5 min, 300 W chamber reactor, 2.45 GHz, 300 W, O_2_-flow = 7 scc min^−1^, Femto BL, Diener, Germany). Afterwards, a layer of silica was deposited on the slides by chemical vapor deposition (CVD) of TEOS in the presence of ammonia. The slides were placed in a desiccator next to two 20 ml vials containing 3 ml TEOS and ammonia each (24 h at room temperature and ambient pressure). Such prepared slides were coated with candle soot collected from a paraffin candle approximately 1 cm above the wick for 40 s (wick height about 0.7 cm, total flame height about 4.5 cm)^43^. The candle-soot template was coated by a second CVD step of TEOS as described above followed by sintering of the slides at 550 °C in air for 3 h (VKM-22, Linn High Therm GmbH, Germany). Finally, the slides were hydrophobized by CVD of a fluorosilane (PFOTS, 100 μl in a 20 ml vial). The slides and the vial were placed next to each other in a desiccator for 3 h at 25 mbar. Residual fluorosilane was removed at 80 °C at 100 mbar for 3 h.

### Fabrication of millimetric hydrogel drops

We prepare soft, medium, and stiff PAAm hydrogel drops by varying the concentration of monomer and crosslinker, leading to elastic modulus values of *E* = 2, 25, and 320 kPa. The modulus is measured by shear rheology (Supplementary Fig. 6). Since the water content is not independent of the modulus, we measured the water fraction by weight, defined as %H_2_O = (*w*_wet_−*w*_dry_)/*w*_wet_, where *w*_wet_ is the weight under equilibrium swelling in water and *w*_dry_ is the dry weight. These are %H_2_O = 97, 94, and 72 for the 2, 25, and 320 kPa hydrogels, respectively.

Since the reaction is oxygen sensitive, we exploit glucose and glucose oxidase as an oxygen scavenger to prevent oxygen inhibition^44^. AAm, BAAm, Irgacure 2959, 500 mg ml^−1^ glucose in water, and 200 μl H_2_O were mixed in an Eppendorf tube and sonicated for 5 min until the components were dissolved. To obtain different hydrogel modulus values, the following table gives the fraction of each of the components added. Table 1

**Table 1:** Hydrogel fabrication compositions

	Acrylamide	BAAm	Irgacure 2959	Glucose	GOx
320 kPa	300	30	5	25	0.02
25 kPa	60	5	5	25	0.02
2 kPa	60	1	5	25	0.02

Fractions of each component in the solution for the different hydrogels. All values are given in mg ml^−1^

Glucose oxidase solution was added and 10 μl drops were immediately dispensed onto the superamphiphobic, 3-well concavity slides. The drops were crosslinked by UV irradiation for 10 min at a light intensity of 3–10 mW cm^−2^ (UV-A LQ 400, Dr. Gröbel UV-Elektronik GmbH, Germany). The crosslinked drops were rinsed off the slides and stored in excess water for at least 48 h before heating experiments.

### Determination of water fractions

Six drops of each type were measured individually by placing swollen drops in separate vial caps and weighed. The drops were dried under vacuum for 48 h at room temperature and then reweighed to calculate the water content.

### Heat-induced jumping and bouncing

Tungsten sheets were clamped between two electrodes connected to a transformer, providing direct current at low voltage. A controlled current of 105 A was applied to the sheet. Individual hydrogel drops were placed on the sheet at room temperature. Hydrogel drops were observed by a high-speed camera (Photron) at 2000 frames per second. Time-temperature profiles of the tungsten sheets were recorded perpendicular with respect to the sheets using an IR-camera to determine the temperature–time profile (VarioCAM HD head, Infratec GmbH, Germany).

### Mechanical characterization

The moduli are determined by both shear rheology measurements and supported by impact experiments on low-adhesion surfaces (Supplementary Note 4). For the stiff hydrogels, deformations were small for impact experiments, and thus were only measured by bulk shear rheology. Samples were prepared in a polystyrene petri dish with a thickness of 0.7 mm. Disk specimens were stamped with a size ranging from 7 to 15 mm. Rheology measurements were taken on a TA Instruments Discovery Hybrid rheometer with roughened surfaces and a minimal amount of super glue.

### Roughness characterization of tungsten sheets

The roughness of the tungsten sheets was measured by atomic force microscopy (Supplementary Fig. 2). The RMS roughness is 15 nm and the maximum roughness scale is 195 nm, measured over 300 µm^2^. The measurements were made in tapping mode using a JPK Nanowizard atomic force microscope in air mounted with an Olympus silicon cantilever (model OMCL-AC240TS-R3, 70 kHz frequency, 2N/m stiffness). The size of pits on the surface were determined by SEM images and image analysis by ImageJ.

### Preparation of hydrophobic and superhydrophobic surfaces

Hydrophobic surfaces were prepared by dissolving amorphous fluoropolymer (2 wt%) in FC-770 fluorocarbon solvent. Tungsten substrates were dipped into the solution, removed and then dried at 150 °C over 4 h to remove residual FC-770. Superhydrophobic surfaces were prepared by doctor blade coating TiO_2_ paste onto tungsten surfaces. All organic molecules were removed by sintering at 500 °C for 1 h, leaving a mesoporous TiO_2_ nanoparticle film. The surface was modified with trichloro(1 H,1 H,2 H,2H-perfluorooctyl)silane by CVD for 2 h under vacuum and then coated with amorphous fluoropolymer by dip-coating (0.5 wt%).

### Data availability

The data obtained and analyzed that support the findings of this study are available upon reasonable request.

## Electronic supplementary material

Supplementary Information

Description of Additional Supplementary Files

Supplementary Movie 1

Supplementary Movie 2

Supplementary Movie 3

Supplementary Movie 4

Supplementary Movie 5

Supplementary Movie 6

Supplementary Movie 7

Supplementary Movie 8

Supplementary Movie 9

Supplementary Movie 10
