# Supplementary material for: Spontaneous jumping, bouncing and trampolining of hydrogel drops on a heated plate
Source: Nat Commun. 2017 Oct 13;8:905. doi: 10.1038/s41467-017-01010-8 (PMC5640668; doi:10.1038/s41467-017-01010-8)
Supplement: Supplementary file 1 — Supplementary Information [file 41467_2017_1010_MOESM1_ESM.pdf]

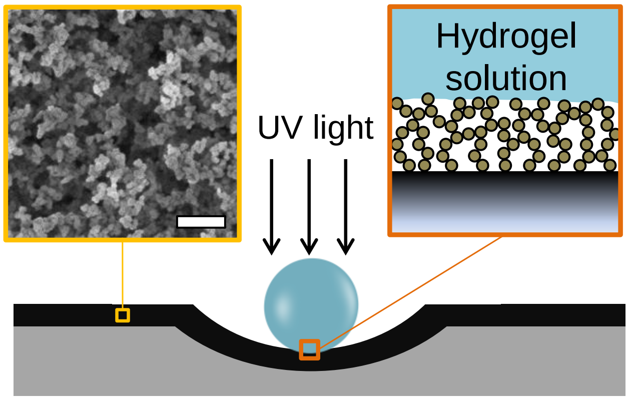

Supplementary Figure 1 | Preparation of hydrogels. Fabrication of millimetric hydrogel drops by using soot-templated superamphiphobic surfaces. Inset: SEM image of the surface (left) and a sketch of the substrate-drop contact area (right). Scale bar: 1 µm.

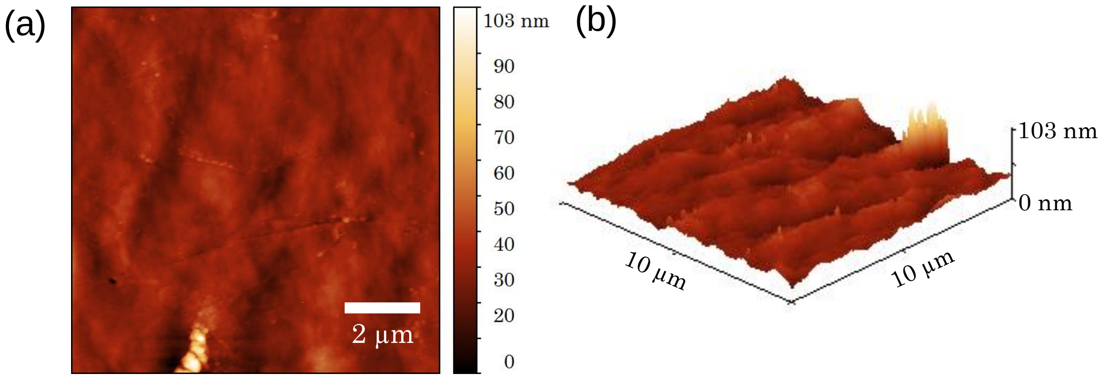

Supplementary Figure 2 | Surface roughness characterization. Examples of (a) 2D and (b) 3D AFM scans for measuring roughness of the tungsten sheet. The RMS roughness averaged over three 100 μm^2 areas is measured as 15 ± 5 nm. The maximum roughness scale, which is taken as the maximum peak to the valley, is 195 ± 89 nm.

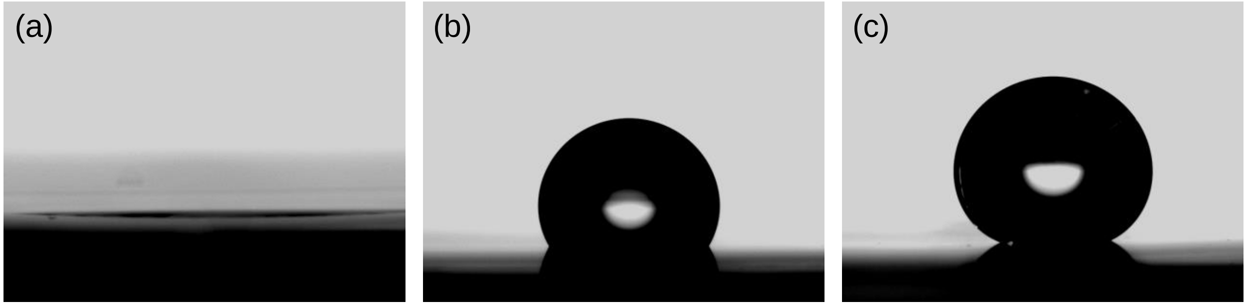

Supplementary Figure 3 | Surface wettability characterization. Optical images of a 5 microliter water drop on (a) tungsten, (b) PTFE-coated tungsten and (c) PTFE-coated TiO_2 surface. The contact angles are less than 5°, $118 \pm 2°$ and $154 \pm 4°$, respectively.

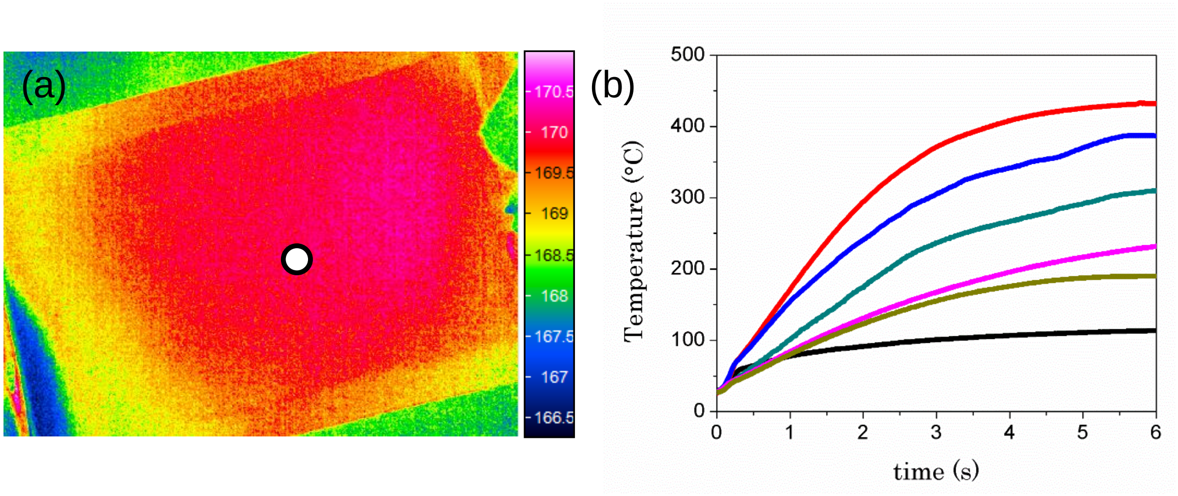

Supplementary Figure 4 | Time-temperature profiles. (a) An example image taken from the infrared camera approximately 1s after turning on the current. The white circle denotes the center position of the measurement, which is where the drops were positioned. (b) A profile of the temperature as a function of time for 45, 65, 75, 90, 100 and 105 Amp settings, which are taken at the center of the tungsten sheet.

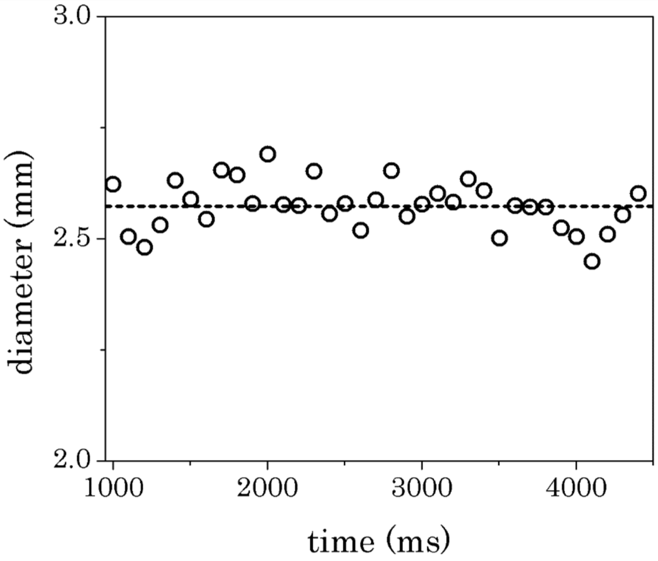

Supplementary Figure 5 | Mass loss characterization during heating. A plot of hydrogel diameter vs. experimental time for the bouncing hydrogel drop in Figure 4a of the main text. The size of the hydrogel does not change in these 4.5 s within our resolution.

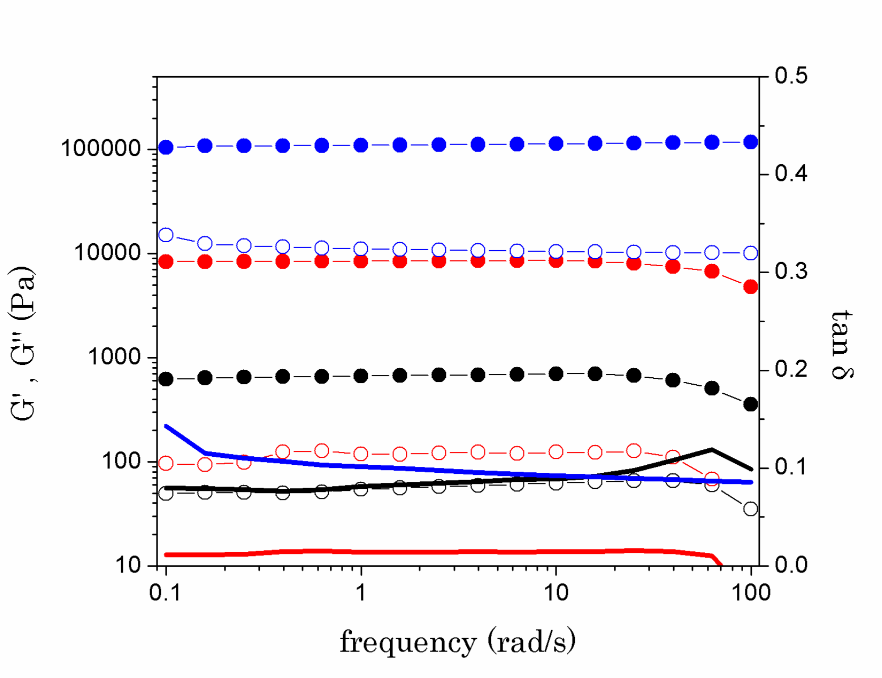

Supplementary Figure 6 | Mechanical characterization of hydrogels. Mechanical measurements by shear rheology of the hydrogels. Filled data represent storage modulus, unfilled data are loss modulus, and solid lines are tan delta. The blue, red and black data sets are for the 320 kPa, 25 kPa and 2 kPa hydrogels, respectively.

Supplementary note 1: Energy required to break the meniscus. To estimate the work required to remove the hydrogel from the surface, we consider a sphere of radius R with a liquid meniscus of volume V having a constant liquid volume. Constant volume is assumed because on the timescale of jumping, the liquid does not significantly evaporate nor flow into or out of the hydrogel. For small menisci ($a \ll R$) the force versus distance is given as:[1]

$$F = 2\pi\gamma R(1+\cos\Theta)\left(1-\frac{D}{\sqrt{\frac{V}{\pi R}+D^2}}\right) \quad (1)$$

Here, Θ is the contact angle formed by water on the substrate; the contact angle on the hydrogel drop is assume to be zero. The work required to pull the sphere from zero distance to infinite distance is obtained by integration:

$$w = \int_0^\infty F dD = 2\pi\gamma R(1+cos\Theta)\int_0^\infty \left(1-\frac{D}{\sqrt{\frac{V}{\pi R}+D^2}}\right)dD$$

$$= 2\pi\gamma R(1+cos\Theta)\left[D-\sqrt{\frac{V}{\pi R}+D^2}\right]_0^\infty = 2\pi\gamma R(1+cos\Theta)\sqrt{\frac{V}{\pi R}} \quad (2)$$

The volume of the meniscus can be approximated by

$$V = \pi a^2 h - \frac{\pi h}{6}(3a^2+h^2) \quad (3)$$

where h is the thickness of the meniscus at its periphery. For geometric reasons $h \approx \frac{a^2}{2R}$ leading to

$$V = \frac{\pi a^4}{2R} - \frac{\pi a^2}{12R}\left(3a^2+\frac{a^4}{4R^2}\right) = \frac{\pi a^4}{2R}\left(1-\frac{1}{2}-\frac{a^2}{24R^2}\right) \approx \frac{\pi a^4}{4R} \quad (4)$$

Then by substitution we find an estimation of the work as

$$w = 2\pi\gamma R(1+cos\Theta)\sqrt{\frac{\pi a^4}{4\pi R^2}} = \pi\gamma(1+cos\Theta)a^2 \quad (5)$$

Supplementary note 2: Bubble expansion into the hydrogel. In the burning regimes, bubbles are often observed to expand into the gel. For the 2 kPa hydrogel, the polymer density is lower compared the 25 kPa hydrogel and thus is burned away more quickly. This breaks the interface and the pressure in a bubble can escape. The interface continues to be burned away and the drop starts bouncing. For the 25 kPa hydrogel, bubbles grow into the gel and remain for a longer time, on the order of seconds (Supplementary Figure 7, Supplementary Movie 10). Similar to the 2 kPa hydrogels, once the interface is burned away, the drop is released and starts bouncing.

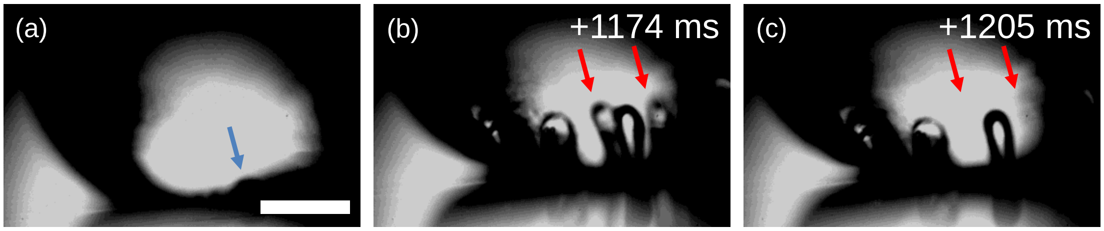

Supplementary Figure 7 | Bubble formation at an adhered interface. Time snapshots of the bubbles expanding to a 25 kPa hydrogel drop. Bubbles can be seen to (a) start to form into the gel (blue arrow) and after ≈1 s, bubbles grow rather large. At one point, the interface starts to break and the pressure in the bubble can be released at the interface. This is clearly illustrated by the bubbles highlighted by (b) the red arrows (1174 ms), which then (c) collapse and disappear (1205 ms). Scale bar: 0.5 mm.

For a bubble to enter the hydrogel, adhesion must be sufficiently high compared to the elastic energy penalty of bubbles expanding into the drop. To provide an idea of the pressures in the system, the critical pressure for a bubble to push into a gel can be approximated by the cavitation equation given by Crosby and coworkers as:[2]

$$P_c = \frac{2\gamma}{r_{\mathrm{pit}}} + \frac{5E}{6} \qquad (6)$$

Here r_{pit} is taken as the average size of a surface pit. By 2D SEM images, the average pit size of our tungsten surfaces is in the range of $r_{\mathrm{pit}} = 270 \pm 170$ nm. Our tungsten surfaces

have an RMS roughness of 15 ± 515 nm and the maximum roughness scale is 195 ± 89 nm, measured by AFM across 300 μm². Then for a 25 kPa hydrogel, $P_c \approx 450$ kPa. When the local interface can sustain pressures greater than P_c, bubbles will expand into the hydrogel drop. When the local interface cannot sustain those pressures, the bubble collapses and the vapor is released through an interfacial crack. This is confirmed by observation of bubbles, which have already grown into the hydrogel, collapsing (Supplementary Figure 7, red arrows). However it must be noted that the adhesion is complicated by its dependence on the hydrogel-tungsten area of contact, the density of molecules at the interface, the dwell time prior to heating, and especially the rate at which the interface is being burned to locally form zero-adhesion areas.

Supplementary note 3: Relevant time scales. Since our experiments are fast, it is helpful to consider the characteristic time scales of different phenomena to provide an intuition of their importance (the evaporation of the drop and the temperature distribution). Calculating the mass loss of an evaporating water drop provides an order of magnitude approximation of the time required for a drop to evaporate. For a spherical approximation, the evaporation time is given as:

$$\tau_{\mathrm{evap}} = \frac{\rho R_{\mathrm{gas}} T R_{\mathrm{gel}}^2}{DM(P_0 - P)} \tag{7}$$

where R_{gas} is the gas constant, T is the temperature in Kelvin, D is the diffusion coefficient and M is the molar mass. Then for $T = 175°\mathrm{C}$, $R = 1.25$ mm, $P_0 = 890$ kPa and $D = 20 \times 10^{-9}$ m² s⁻¹,[3] we obtain $\tau_{\mathrm{evap}} = 1815\,\mathrm{sec} \approx 30\,\mathrm{min}$. This is significantly slower than the several second time scale of the experiment, which is consistent with our measurement of a constant hydrogel size during bouncing (Supplementary Figure 5). The temperature distribution rate scales with the thermal diffusivity, κ_{T}. Then the time for thermal equilibrium to occur will follow $\tau_{\mathrm{T}} = 2R_{\mathrm{gel}}^2/\kappa_{\mathrm{T}} \approx 20$ s. In reality, this time may differ slightly because the thermal diffusivity of water $\kappa_{\mathrm{T}} = 1.4 \times 10^{-7}$ m² s⁻¹ at 25°C) will be higher at higher temperature (but similar order),[4] while contact with the substrate will require more heat flow. Regardless, this illustrates that our experiments are of a

similar order to the thermal equilibrium time while the pressure and evaporation have negligible relevance.

Supplementary note 4: Supporting measurement of modulus for 2 and 25 kPa hydrogel drops by impact. For soft gels, measuring the modulus that correctly reflects the drop is challenging because variations arise during the preparation as compared to macroscopic testing specimens. Moreover, measuring the modulus of such soft, brittle gels can be difficult practically. Therefore, in addition to the shear rheology measurements presented in Supplementary Figure 6, we also measured the elastic modulus of hydrogel drops by letting them impact on a flat surface and measured the deformation δ and the impact velocity v via high speed video microscopy. This provides a second method to confirm the measurements on macroscopic samples. By assuming that Hertzian collisions correctly describe the impact, one can extract the modulus as:

$$E_{\mathrm{Hertz}} = \frac{45mv^2}{64R^{1/2}\delta^{5/2}} \tag{8}$$

We first released hydrogel drops onto the tungsten surfaces at room temperature. However, high adhesion and capillary forces keep the drops from rebounding. Even on a Teflon surface, the hydrogel drops are barely able to overcome capillary adhesion, if at all. By releasing hydrogel drops onto a low-adhesion, soot-templated, superamphiphobic surface, we are able to observe large, near-adhesionless rebounds. We consider these impacts as elastic, such that Supplementary Equation 8 is suitable. For the softest gel, we find the modulus to be $E \sim 4.8 \pm 1.5$ kPa and for the medium gel we find $E \sim 27 \pm 4$ kPa. These are in reasonable agreement with the measurements by rheology.

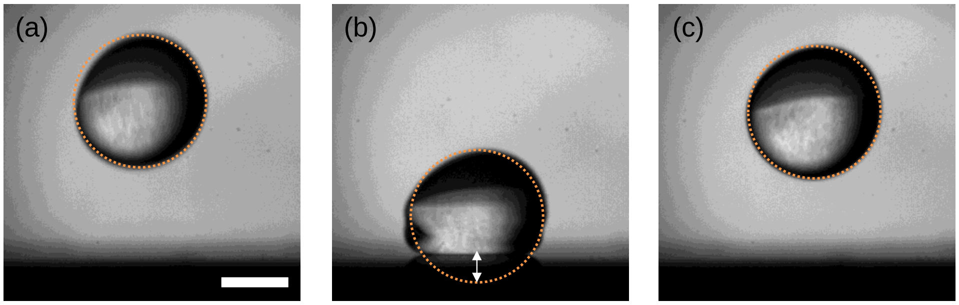

Supplementary Figure 8 | Images of drop impact measurements. An example of a ~2 kPa hydrogel drop impacting an unheated, low-adhesion, soot-templated surface (a) before, (b) during and (c) after impact. The white arrow illustrates the normal deformation, $\delta \approx$ 620 μm. Scale bar: 1.5 mm.

As a test for the effect of surface tension, we add an additional surface term to the energy balance as $U_{\mathrm{kin}} = U_{\mathrm{Hertz}} + U_{\mathrm{surf}}$ where $U_{\mathrm{surf}} = 2\gamma\Delta A$. Here A is the surface area of the drop and γ is the surface tension, which we assume is constant and that of water (i.e., $\gamma =$ 0.072 N/m). We approximate A before contact by assuming a sphere and during contact by fitting an ellipse. For the drop shown in Supplementary Figure 8, the elastic energy is on the order of ~800 nJ while the surface energy is on the order of ~25 nJ. Since it is more than an order of magnitude lower, and is only about 3% of the total energy, we neglect it here. As a confirmation, we can include the surface term into the modulus determination as

$$E_{\mathrm{Hertz},\gamma} = \frac{45(mv^2 - 2\gamma\Delta A)}{64R^{1/2}\delta^{5/2}} \quad (9)$$

For the drop shown in Supplementary Figure 8, $E_{\mathrm{Hertz}} = 3.2$ kPa while $E_{\mathrm{Hertz},\gamma} = 3.1$ kPa.

Supplementary References

1. Butt, H. J. & Kappl, M. Normal capillary forces. *Adv. Colloid Interface Sci.* **146,** 48–60 (2009).

2. Kundu, S. & Crosby, A. J. Cavitation and fracture behavior of polyacrylamide hydrogels. *Soft Matter* **5,** 3963–3968 (2009).

3. Krynicki, K., Green, C. D. & Sawyer, D. W. Pressure and temperature dependence of self-diffusion in water. *Faraday Discuss. Chem. Soc.* **66,** 199 (1978).

4. Abramson, E. H., Brown, J. M. & Slutsky, L. J. The thermal diffusivity of water at high pressures and temperatures. *J. Chem. Phys.* **115,** 10461 (2001).
