# Supplementary material for: Spontaneous jumping, bouncing and trampolining of hydrogel drops on a heated plate
Source: Nat Commun. 2017 Oct 13;8:905. doi: 10.1038/s41467-017-01010-8 (PMC5640668; doi:10.1038/s41467-017-01010-8)
Supplement: Supplementary file 2 — Description of Additional Supplementary Files [file 41467_2017_1010_MOESM2_ESM.pdf]

Description of Additional Supplementary Files

File Name: Supplementary Movie 1
Description: Fabrication of hydrogel drops by rolling drops of monomer/crosslinker solution on a candle-soot templated surface

File Name: Supplementary Movie 2
Description: A 25 kPa hydrogel drop displaying meniscus jumping

File Name: Supplementary Movie 3
Description: Zoom-in of rapid meniscus evaporation driving spontaneous hydrogel jumping

File Name: Supplementary Movie 4
Description: An initially stuck 2 kPa hydrogel drop. The polymer adhering the drop to the substrate burns away as the plate is continuously heated, followed by bouncing and trampolining

File Name: Supplementary Movie 5
Description: A 320 kPa hydrogel drop displaying meniscus jumping

File Name: Supplementary Movie 6
Description: Sliding/rolling of 25 kPa hydrogel drop on a PTFE-coated tungsten sheet

File Name: Supplementary Movie 7
Description: A 25 kPa hydrogel being heated on a superhydrophobic surface, leading to the Leidenfrost regime

File Name: Supplementary Movie 8
Description: A 25 kPa hydrogel drop jumping, bouncing and trampolining

File Name: Supplementary Movie 9
Description: A 25 kPa hydrogel showing super short ($\lesssim$ 0.5 ms) contact time

File Name: Supplementary Movie 10
Description: A 25 kPa hydrogel with bubble expansion into the solid
